# Treatment of the Mal de Debarquement Syndrome: A 1-Year Follow-up

**DOI:** 10.3389/fneur.2017.00175

**Published:** 2017-05-05

**Authors:** Mingjia Dai, Bernard Cohen, Catherine Cho, Susan Shin, Sergei B. Yakushin

**Affiliations:** ^1^Department of Neurology, Icahn School of Medicine at Mount Sinai, New York, NY, USA; ^2^Department of Neurology, NYU Langone Medical Center, New York, NY, USA; ^3^Department of Otolaryngology, NYU Langone Medical Center, New York, NY, USA

**Keywords:** vestibular, adaptation, rocking, swaying, bobbing, velocity storage, sea legs, disembarking syndrome

## Abstract

The mal de debarquement syndrome (MdDS) is a movement disorder, occurring predominantly in women, is most often induced by passive transport on water or in the air (classic MdDS), or can occur spontaneously. MdDS likely originates in the vestibular system and is unfamiliar to many physicians. The first successful treatment was devised by Dai et al. (1), and over 330 MdDS patients have now been treated. Here, we report the outcomes of 141 patients (122 females and 19 males) treated 1 year or more ago. We examine the patient’s rocking frequency, body drifting, and nystagmus. The patients are then treated according to these findings for 4–5 days. During treatment, patients’ heads were rolled while watching a rotating full-field visual surround (1). Their symptom severity after the initial treatment and at the follow-up was assessed using a subjective 10-point scale. Objective measures, taken before and at the end of the week of treatment, included static posturography. Significant improvement was a reduction in symptom severity by more than 50%. Objective measures were not possible during the follow-up because of the wide geographic distribution of the patients. The treatment group consisted of 120 classic and 21 spontaneous MdDS patients. The initial rate of significant improvement after a week of treatment was 78% in classic and 48% in spontaneous patients. One year later, significant improvement was maintained in 52% of classic and 48% of spontaneous subjects. There was complete remission of symptoms in 27% (32) of classic and 19% (4) of spontaneous patients. Although about half of them did not achieve a 50% improvement, most reported fewer and milder symptoms than before. The success of the treatment was generally inversely correlated with the duration of the MdDS symptoms and with the patients’ ages. Prolonged travel by air or car on the way home most likely contributed to the symptomatic reversion from the initial successful treatment. Our results indicate that early diagnosis and treatment can significantly improve results, and the prevention of symptomatic reversion will increase the long-term benefit in this disabling disorder.

## Introduction

The mal de debarquement syndrome (MdDS) is a maladapted vestibular movement disorder characterized by continuous rocking, swaying, or bobbing that can follow a cruise, flight, or ground transport (the classic MdDS) and can also occur spontaneously (1–7). The continuous oscillating movements are often accompanied by additional symptoms, such as bouncing while walking, intolerance to lights and noises and crowds, impaired cognition, lack of mental clarity (i.e., brain fog), blurry vision, anxiety, depression, and lethargy. These symptoms are generally relieved temporarily while riding in a car or a plane, but return promptly when the ride ends and can persist for months or years. At present, MdDS is not widely recognized by the medical community, and, until recently, there was no effective treatment. While MdDS has been classified as a rare, disabling disease, with the wide interest in cruises, there is a steady stream of patients who contract this illness.

Experimental human and animal studies have provided insight into the neural structures and mechanisms involved in producing this syndrome. MdDS likely originates from the vestibular system since people without vestibular function have the same or better postural stability after 200 miles of sea voyage (8), and the syndrome does not occur in monkeys with short-time constants of the vestibular-ocular reflex (VOR), i.e., in animals without velocity storage (9). Thus, MdDS does not occur in subjects with severe vestibular damage. VOR moves the eyes to stabilize gaze in space during rotation of the head around any axis. Head movements in yaw produce horizontal eye movements. Pitch head movements produce vertical eye movements, and roll head movements produce ocular torsion (10). Thus, simple head roll along the naso-occipital axis only produces torsional eye movements and/or nystagmus without a component in pitch (11, 12). There are situations, however, where the VOR occurs across more than one axis. For example, if the head rolls while the body is rotating in yaw, there is activation of the semicircular canals in both pitch and roll. As the head is turned, the vertical canals are brought into the plane of rotation, producing vertical nystagmus and a strong sensation of tumbling in pitch (9, 13, 14). Similar results were also produced by roll while rotating in cynomolgus monkeys (14). This is an unusual vestibular stimulus that we designate as a cross-axis-coupled stimulus (9) because the third axis of eye movement is produced by a cross-product of the other two axes (15) and has been used in training astronauts for mitigating motion sickness susceptibility (9, 16–18). A cross-axis-coupled stimulus can alter velocity storage of the VOR (19, 20) to produce persistent, abnormal eye movements (1, 14, 21).

The VOR is composed of three components: yaw, pitch, and roll. All three are the subject of adaptation (22). Adaptation of the VOR can occur in a specific context across different axes (23). Contextual adaptation is long lasting, and the changes induced by 1 h of training can last for several days (24–27). As described in the paragraph above, simultaneous rotation about yaw and oscillation in roll induces oscillation in pitch (9, 14). If a subject is adapted to that context, then, similar to cross-axis adaptation, simple oscillation in roll can induce adaptive compensatory eye oscillations in pitch. This finding became the bases for the hypotheses of the underlying mechanism of MdDS (1). Cruise ships frequently change their course. In addition, the ships are constantly rocking side to side at ≈0.2 Hz (28). Most of the people become motion sick from such a complex stimulus; however, others are well adapted and feel fine while in motion. On disembarkment, however, small head motions, side to side with every step, induce a sensation of gravitational pull forward and backward. When motionless, however, the patient experiences a sensation of constant rocking at ≈0.2 Hz.

We reasoned that if the notion of MdDS caused by maladaptation of the VOR holds true, a reversed VOR stimulus should mitigate or even cure the MdDS symptoms. Based on these findings, a treatment for MdDS was devised that consisted of rolling the head at the frequency of body rocking, while the subjects viewed a slowly moving visual surround. This postulate was tested in 24 MdDS patients employing cross-axis visual coupling (1). The results were promising: 75% of the MdDS patients had significant improvement in a 12-month observation, which was the first successful treatment of this debilitating illness. Since then, 330 MdDS patients have been treated with a similar initial success rate. This report documents the follow-up of 141 of these treated patients who had a year observation, of whom 120 had the classic MdDS and 21 had the spontaneous MdDS.

## Materials and Methods

### Patient Selection

Subjects were screened with a brief interview and an intake form. The inclusion criteria were reports of continuous rocking, swaying, and/or bobbing, which had persisted for at least 3 weeks. Their symptoms were temporarily relieved during car rides (1) and returned immediately after the rides had ended. They had no history of head or neck trauma, Lyme disease, vestibular disease, or major neurological or vascular disorders. For all patients if the oscillating sensation began less than 3 days after a passive motion event, it was designated as the classic form of MdDS. If the oscillating sensation was not preceded by a motion event or it took place more than 3 days after a motion event, it was designated as the spontaneous form of MdDS. The patients were referred by medical doctors, physiotherapists, or former patients, or were self-referred. Many had completed neurologic and otologic workups including MRIs. These studies were generally normal.

### Treatment Procedure

The clinical treatment has been detailed in our previous study (1). A critical aspect was to establish the frequency of the rocking and swaying. Static posturography for stability was performed using a Wii board that measured rocking and sway (1). The dominant oscillating frequency was determined from the power spectra of the recorded center of pressure (COP) (29). If the body movements were not expressed physically, the patients were asked to move their arm attached to an acceleration sensor at the frequency of the internally sensed movement.

During the treatment, the head was rolled from side to side at the measured frequency while they watched the movement of vertical stripes projected onto the wall of an enclosure that surrounded them. The direction of the horizontal optokinetic (OKN) stimulus was predetermined based on the Fukuda stepping test, circular body rotation, or spontaneous horizontal nystagmus that was due to the maladaptation of the VOR (14). In some cases, the direction of OKN could not be determined. If so, an arbitrary direction was tested for ≈30 s to see whether it was effective to improve the symptoms. If the symptoms became worse or there was no effect, the direction of drum rotation was reversed. The patients were treated 10–120 min a day for 4–5 consecutive days. On the first and last day, their balance was measured by posturography, and they were asked to rate their level of MdDS severity and to describe their symptoms.

### Subjective Measures

The levels of subjective severity were reported on a simplified 10-point scale before and after treatment. A score of 10 was the most severe symptoms that they had experienced. A score of 5 was moderate, and they scored 0 if they were completely symptom free. The subjective score was based on the patient’s own experiences as well as being dependent on their initial severity level before the treatment. There has been no unified self-scoring index system, and the self-ratings were used for the percentage change in subjective severity as an assessment of treatment effects.

### Survey Process

The patients were asked to report the status of the MdDS severity and specific symptoms retrospectively at 2 weeks and 3, 6, and 12 months after treatment. The common symptoms reported are listed in Table 1.

**Table 1:** **Percentage of the symptom occurrence**.

Symptoms	Pretreatment	A year later
Rocking	80	42
Swaying	73	44
Bobbing	46	32
Gravity pulling	54	39
Trampoline walking	29	30
Fatigue	62	54
Unstable in crowds	51	60
Brain fog	53	49
Sensitivity to fluorescent lights	51	48
Sensitivity to computer screens	54	40
Anxiety	49	41
Depression	38	23
Fullness of the ears	39	23
Tinnitus	33	27
Fussy vision	37	29
Clumsiness	37	37
Head pressure	28	32

### Assessment of Treatment

A reduction of the symptom severity by more than 50% was deemed a clinically significant improvement or success of treatment. The treatment results of symptom severity were related to the forms of MdDS, duration, age, gender, and posturography.

The displacement of COP over a 20-s period was measured, and the root mean square (RMS) of the postural displacement was computed (30) to compare the postural stability before and after the treatment (1). The total trajectory length (maximum excursion) of the COP deviation was computed over 20 s.

### Statistics

ANOVA and Student’s *t*-tests were used to establish the statistical significance of the findings.

## Results

### Demographics and Attributions

One hundred forty-one patients of 155 who were treated a year or more ago and who responded to our follow-up formed the basis for this report. The average age of the female patients was 49 ± 13 years and for male was 38 ± 13 years. There were 120 classic cases with 100 females and 20 males, a ratio of 5:1. Thus, classic MdDS was more common in women than in men. The spontaneous group was composed of 2 males and 19 females, also a strong female predominance. Since the spontaneous group is small, the majority of the statistical analyses was performed for patients with classic MdDS. In the classic group, there were 56 individuals in whom the MdDS was triggered by a cruise (47%); 30 (25%) by boating; 23 (19%) by air flights; 7 (6%) by car, bus, or train rides; and the others by playing video games, rafting, or scuba diving.

In the spontaneous group, nine patients (43%) could not recall a particular event that preceded their MdDS. One had had classic MdDS that had remitted years ago. The MdDS in eight patients was preceded by a period of stress and anxiety from switching medication, severe headache, surgery, or heavy drinking. In the remaining three patients, one reported that his MdDS was triggered by an episode of benign paroxysmal positional vertigo. A brief motion triggered the other two patients: one due to a sudden drop of a helicopter and the other due to a 360° spin of a vehicle on ice.

### Primary Symptoms

Symptoms associated with MdDS were reported widely and differently, and they are listed in Table 1. Many patients experienced more than one type of motion. The primary symptoms were related to their motion sensation, either observable physically or perceived internally. The most common sensation was rocking, i.e., an oscillation back and forth, recorded or reported in 95% of the patients. Next most common sensations were swaying, i.e., side to side, in 55% and bobbing up and down in 43% of patients. In addition, bouncing while walking, which was labeled as “trampoline walking,” was often a complaint. There were variations in trampoline walking. Some patients felt that they were pushed as if they were walking on an airport moving walkway, others experienced “moon walking” up and down in a slow motion, and the others felt side-to-side bouncing for each step as if they were walking on a floating dock. These bounces could be felt only in some parts of the body, i.e., in the head, trunk, and legs or feet. Besides the motion sensations, some patients also experienced a steady pulling sensation on the body in a particular direction when standing still. This was labeled as “gravity pulling.” Falling backward due to the backward pulling was most common.

### Additional Symptoms

These were mainly secondary to the primary problem of incessant motion. They occurred immediately or days or months after the onset of the MdDS. They included light or sound sensitivity, head pressure, heaviness of the body, brain unclearness (fog), fuzzy vision, claustrophobia, agoraphobia, stress, anxiety, fatigue, insomnia, and depression, and they were not uncommon. The symptoms could be exaggerated by physical or visual motions, hormonal changes, weather changes, stress, and fatigue. The symptoms could also be aggravated by coffee (28%) and alcohol (21%). Some patients had prolonged motion after-sensations (aftereffects), i.e., those that followed a motion or movement (31). The persistent symptoms were incapacitating, and many even were unable to continue working.

About half of the patients developed heightened motion and visual sensitivity. As a result, they experienced motion sickness as well as migraine-like symptoms, such as headache, pulsating head pressure, and woozy and queasy feelings. Most of them, however, did not have a clear history of migraine or motion sickness predating their MdDS.

### Initial Posttreatment Results

The average score of severity for the classic group before treatment was 6.1 ± 1.9. Immediately after treatment, the average score was 2.4 ± 2.1 (*P* < 0.0001, *N* = 120), with a rate of success of 78%. Twenty-three became asymptomatic (19%) after the treatment. In the spontaneous group, the average score of severity before treatment was 6.6 ± 1.8. After treatment, the average score was 2.5 ± 2.8 (*P* < 0.001, *N* = 21), with a rate of success of 48%.

### Postural Instability and Subjective Improvement

**Figure 1:**
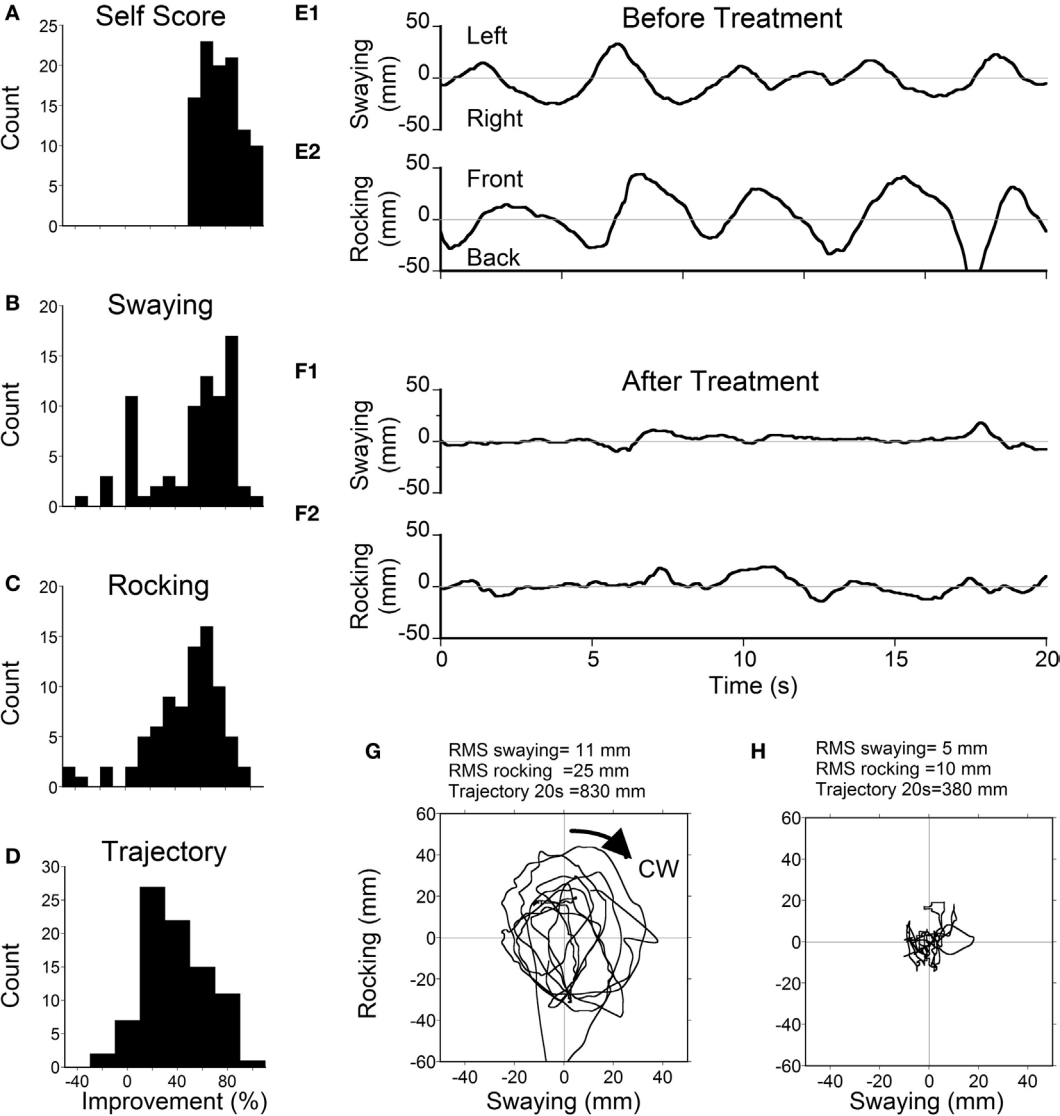
**Scores (A) and posturography (B–D) in all patients with ≥50% self-scored improvement**. **(A)** Histogram of self-scores (median = 75%). **(B)** Histogram of improvement in postural sway (median = 63%). **(C)** Histogram of improvement in rocking (median = 55%). **(D)** Histogram of the total trajectory length (maximum excursion) of the center-of-pressure (COP) deviation (median = 38%). **(E1)** Example of pretreatment sway with a dominant frequency of 0.2 Hz. **(E2)** Pretreatment rocking with a frequency of 0.2 Hz. **(F1)** Posttreatment sway with no specific frequency. **(F2)** Posttreatment rock. **(G)** Pretreatment trajectory of COP. **(H)** Posttreatment trajectory of COP.

Static posturography was recorded before and a week after treatment for 105 patients, and the relationship in improvement between the subjective measure and postural stability was determined for patients who reported >50% improvement. These data are shown in Figure 1. The median value of the self-reported improvement was 75% (Figure 1A), which was better than the median values of improvement in swaying (63%; Figure 1B), rocking (55%; Figure 1C), or maximum body excursion (38%; Figure 1D). These data indicate that postural improvement was concomitant with subjective improvement, but there was not a simple quantitative relationship between the subjective measure and the postural stability. The actual traces of postural frequency were predominantly at 0.2 Hz, which is roughly 90° phase shifted from the sway (Figure 1E1). After treatment, there was no predominant frequency (Figure 1F2). The rocking combined with swaying (Figure 1E1 and 2) formed a circular progression of the trunk around the stationary feet. This circular progression (CW) can be best seen in the trajectory plots of the COP deviation (Figure 1G) recorded by a Wii board. After treatment, the circular progression disappeared together with a smaller COP trajectory (Figure 1H). In these examples, the RMS of sway before treatment was 11 and 5 mm after treatment. The RMS of rocking before treatment was 25 and 10 mm after treatment. The disappearance of a dominant oscillating frequency and the reduction of the RMS of rocking and sway provided objective data supporting the efficacy of treatment. The average rocking frequency for all classic patients was 0.23 ± 0.16 Hz (ranging from 0.02 to 1.0 Hz), which was significantly lower than the sway frequency (0.29 ± 0.17 Hz, ranging from 0.06 to 0.8 Hz, *P* = 0.011). There was no difference in rocking (*P* = 0.46, ANOVA) and swaying frequencies (*P* = 0.44, ANOVA) between age groups.

### One-Year Follow-up

#### Symptoms Reported from Follow-ups

Table 1 shows the percentage of all patients who experienced the spontaneous symptoms before the treatment and thereafter for a year. The patients did not specifically grade the severity for each of the symptoms. A year later, 38, 29, and 14% of patients who initially experienced rocking, swaying, or bobbing were free of these symptoms respectively. Meanwhile, 15% had recovered from gravity pulling. For some, although the symptoms were still lingering a year later, they were much milder and infrequent compared to the pretreatment period.

**Figure 2:**
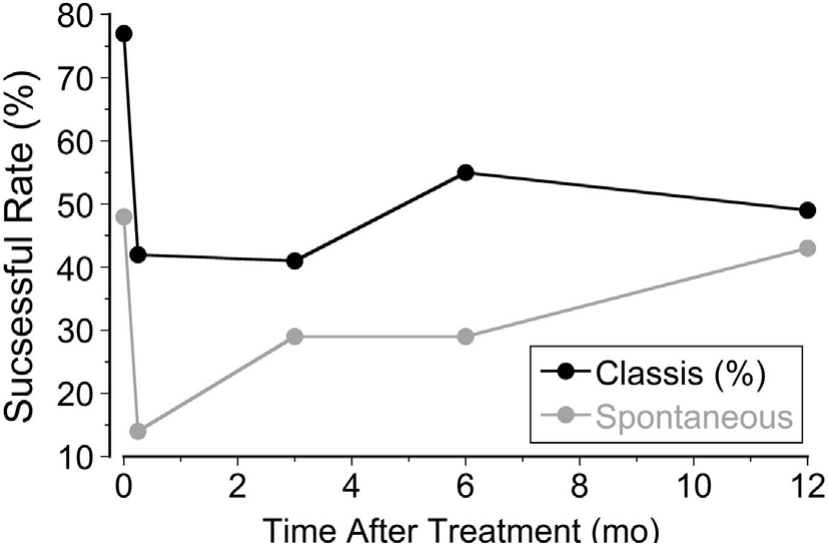
**Percentage of patients improved at week 1, week 2, month 3, month 6, and month 12 after treatment**.

In the classic group, there was a significant setback 2 weeks after treatment. The rate of subjective improvement dropped to 42% from the initial 78%. A year later, the rate increased to 53% (Figure 2), and 32 patients did not experience any symptoms (27%). There was also a significant setback in the spontaneous group. The rate dropped to 14% at week 2 from the initial 48%. It climbed back to 48% a year later, however (Figure 2). There was no difference in the improvement rate between classic and spontaneous groups at 1 year. Interestingly, nine classic patients and three spontaneous patients, who did not improve initially, were significantly improved 1 year later.

#### Difference between Genders

**Figure 3:**
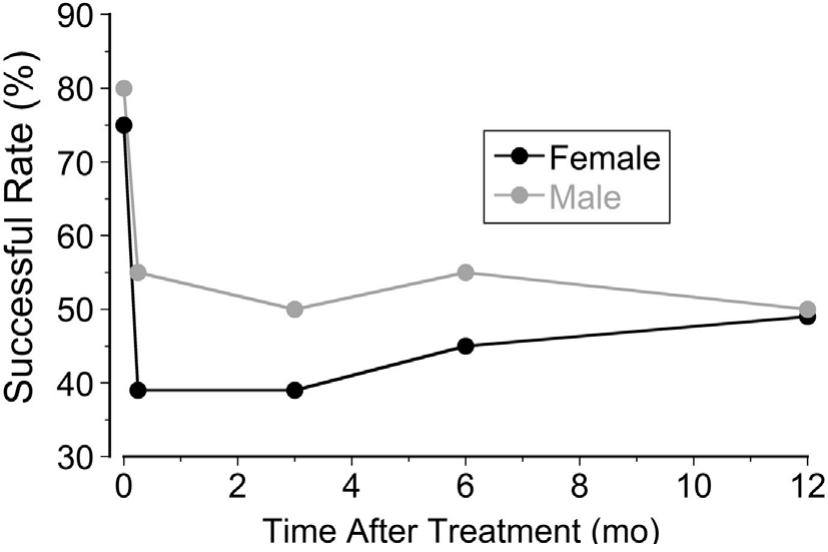
**Improvement rates in female and male patients at week 1, week 2, month 3, month 6, and month 12 after treatment**.

The most striking difference was the heavy predominance of the MdDS in females, in both the classic and spontaneous groups. Effects of treatment were similar, however, in both genders (80% for male and 75% for female). The success rate dropped to 55% in male patients and 53% in female patients a year after treatment (Figure 3). Small gender differences were noted at week 2, and the improvement rate dropped to 39% in females but less in males (55%).

#### Effects of Symptom Duration on Treatment

**Figure 4:**
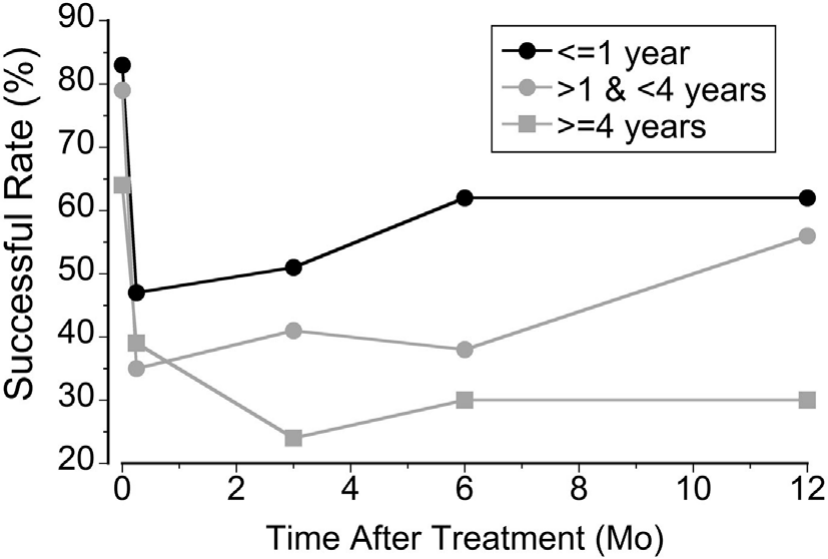
**The relationship between the rate of success and the duration of mal de debarquement syndrome at week 1, week 2, month 3, month 6, and month 12 after treatment**.

The 120 classic patients were examined for the effects of symptom duration of MdDS on treatment (Figure 4). The patients were divided into three groups based on the duration of the illness; 1 year or less (Group 1, *N* = 53), more than 1 year to 3 years (Group 2, *N* = 34), and more than 3 years (Group 3, *N* = 33). The initial rate of success was higher in Group 1 (85%). This was followed by Group 2 (79%) and Group 3 (64%). The success rate decreased to 47% (Group 1), 35% (Group 2), and 39% (Group 3) at week 2 when they returned home. There was a recovery in Group 1 (62%) and Group 2 (56%) at a year but not in Group 3 (30%). There was no difference between Groups 1 and 2 over a year (*P* = 0.07, repeated measures, ANOVA), but there was a significant difference between Groups 1 and 3 (*P* = 0.0003) and Groups 2 and 3 (*P* = 0.013). Thus, in general, the treatment was less successful for those with MdDS for more than 3 years. There were exceptions, however. Seven patients whose history was greater than 9 years (from 9 to 27 years) had a better outcome than Group 3 as a collective. The initial rate was 71% that declined to 57% after week 2 but still held at 43% after a year.

#### Effect of Age on Treatment

**Figure 5:**
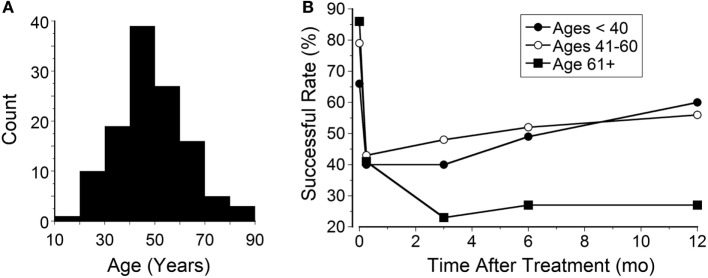
**(A)** Age distribution of patients with classic mal de debarquement syndrome (mean 48 ± 14 years). **(B)** The success rate of the three groups over a year.

The average age for patients with classic MdDS was 48 ± 14 years (Figure 5A). This was similar to the mean age in our prior study (1). These patients were divided into three age groups: less than 40 years (Group 1, *N* = 28), 40–60 years (Group 2, *N* = 70), and greater than 60 years (Group 3, *N* = 22). The initial effect was better in the oldest patients (Group 3, 86% improvement). This was followed by Group 2 (79%) and then by Group 1 (66%). The initial rate of success dropped to 42% at 2 weeks for all three groups (Figure 5B). There were no significant differences between Groups 1 and 2 in the success rates over a year after the initial improvement at week 1 (Figure 5B; *P* = 0.99, ANOVA). Despite the higher initial success rate in patients who were older than 60 years, they were not able to maintain this improvement. Their rate of improvement declined to below that in the younger groups (Figure 5B; *P* < 0.0004). Groups 1 and 2 recovered to 53% and Group 3 did not improve over a year (Figure 5B).

#### Recurrent MdDS

Recurrent episodes of MdDS were experienced by 23% of the classic patients (25 of 120). They experienced a relapse due to another cruise, boating, a long haul flight, or an extreme stress, or they had a slow regression after a period of remission. There was no difference in age or gender between patients who had recurrent episodes or a single episode of MdDS. The initial improvement in these two groups was approximately the same (67% in patients with recurrent episodes versus 72% in patients with a single episode). A year later, 63% of patients with recurrent episodes had improved, but only 50% of patients with a single episode were better.

#### Progression over a Year between Long-term Successful and Unsuccessful Groups

**Figure 6:**
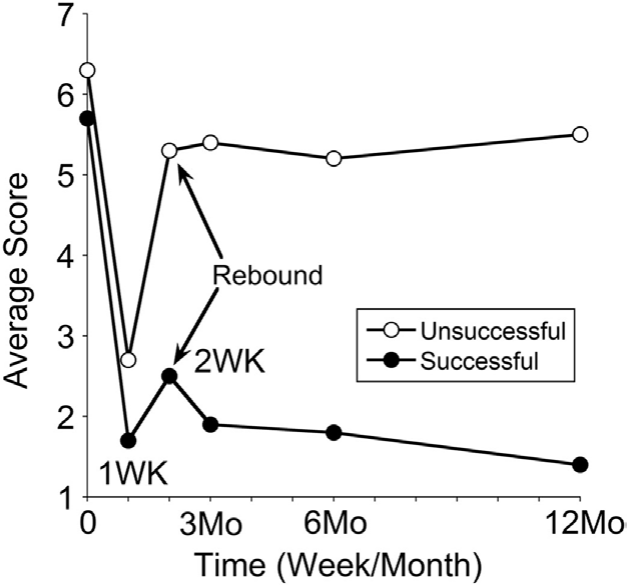
**Progression in severity scores over a year in treatment-successful and treatment-unsuccessful groups**. Time 0 is pretreatment, followed by posttreatments at week 1, week 2, month 3, month 6, and month 12. The symptom rebound at week 2 is indicated by arrows. The SDs of the data set were not shown, which are from 1.0 to 2.0.

The classic patients were divided into two groups: those in whom 50% improvement had occurred in 12 months and those with a lesser successful rate. Sixty-two were successful, and 58 were unsuccessful. Their progression over a year is shown in Figure 6. The pretreatment severity score for the successful group was not different from that of the unsuccessful group (5.7 ± 1.9 versus 6.3 ± 1.8; *P* = 0.08). There was a smaller rebound of the average score in the successful group from 1.7 ± 1.5 at week 1 to 2.5 ± 2.1 at week 2 (47% increase). There was a larger rebound in the unsuccessful group from 2.7 ± 2.2 at week 1 to 5.3 ± 2.4 at week 2 (96% increase). Despite the small rebound at week 2 in the successful group, the improvement by more than 50% was maintained at any given time of the year. These data indicate that when the rebound at week 2 was small, the improvement would last, and *vice versa*. Of note is that some of our successful patients had an additional motion sickness treatment that desensitized their motion and visual sensitivity (32), which in turn may actually have reduced the chance of reversion. More study is needed to investigate the relationship between the visual–motion sensitivity and the reversion of MdDS.

While there was no such 50% improvement over a year in the unsuccessful group after the larger rebound at week 2, however, there was a statistically significant reduction in severity between the pretreatment and at month 12 (*P* = 0.005). These accompanied their general feelings of less and milder symptoms.

## Discussion

### Summary of Findings

Using a full-field visual stimulus while the head was rolled the severity of MdDS symptoms was successfully improved by ≥50% immediately after the treatment in 78% of 120 classic patients and in 48% of 21 spontaneous patients. The improvement was also shown in posturography findings (Figure 1). In the classic cases, the initial improvement rate of subjective findings regressed to 42% over 2 weeks upon returning home, but rose to 52%, 12 months later. The success rate of spontaneous cases fell to 14% 2 weeks after treatment, but then rose to 48%, 12 months later (Figure 2). For patients who did not have a 50% reduction, there was still a statistically significant reduction in subjective symptoms a year later compared to the pretreatment level (*P* = 0.005). There was a marked preponderance of females with MdDS, but there was essentially no difference in the response to treatment between genders (Figure 3). There was also the same tendency for a reduced rate of success in patients who had had the MdDS for long periods (Figure 4), or they were of an advance age (Figure 5). This was not universal, however. There were three patients with more than 20 years of symptom duration who were treated successfully. Thus, we were able to essentially reproduce the beneficial results of treatment for MdDS, a condition that had had no successful treatment prior to our earlier study (1). The medical community is generally unaware that the MdDS is a characteristic set of signs and symptoms, and the patients who came to us most frequently had a full medical workup with many medical tests, costing thousands of dollars by the time that these patients were diagnosed and referred. The knowledge that there is such a syndrome and further that it can be successfully treated would be a benefit to both patients and the medical system.

There was a significant reversion in improvements when patients traveled back home at week 2, more in women than in men (Figure 2). There are many reasons to account for the reversion. The major factors most probably were the long duration of traveling and anxiety. The rate of reversion when traveling home was higher in this study (36%) than in the previous study, in which only about 22% of successfully treated patients had a regression of symptoms (1). The reason for this difference is not clear, but in the previous study, most patients lived nearby and did not have to travel long distances to return home. More than half of the patients also developed heightened visual and motion sensitivity following the onset of the MdDS. When their oscillating symptoms were treated, there were still a portion of patients who were susceptible to visual and motion stimuli, such as intolerance to fluorescent lights, flickering screens, and busy patterns. Some of them reported that their symptoms had reverted when they were exposed to patterned hallway or extreme lighting conditions. The subjects were asked to take a small dose of a benzodiazepine when traveling home, but it did not always produce the desired result.

One of the interesting outcomes from this follow-up is that when there was no or less reversion, the improvement was sustainable, and *vice versa* (Figure 6). Thus, the avoidance of the reversion in the first 2 weeks could be vital to retain the treatment effects. On the other hand, if this setback in the first 2 weeks could be reverted back to the week 1 posttreatment level using an effective home-based treatment, it might help those patients with reversions.

### The Neural Basis of the MdDS

Mal de debarquement, or sea legs, is a natural phenomenon after some sea voyages, ordinarily only lasting for up to 24 h, even after long voyages (33, 34). It can also occur from other transport modalities. This is different from the MdDS that can last for weeks, months, or years (1, 2, 4, 35). The neural basis for the MdDS was shown in humans and monkey experiments to be produced by a combination of roll with rotation (14, 21) that caused a deviation of the yaw axis eigenvector from gravity, i.e., the axis around which the eyes rotate in upright and tilted positions.

The neuronal activity responsible for MdDS is probably in the velocity storage integrator located in the vestibular-only (VO) neurons in the medial and superior vestibular nuclei (36). Monkeys without velocity storage and very short VOR time constants do not develop abnormal responses to roll while rotating, and the same is true for humans without velocity storage, confirming that the MdDS is a vestibular phenomenon, arising in the brainstem. The VO neurons are GABA_b_-ergic, and their axons cross the brainstem (37, 38), and they send activity into the reticulospinal and vestibulospinal pathways (39). Thus, they could provide the vestibulospinal activity to produce the rocking, swaying, and bobbing to the body and limbs. VO neuron activity can also reach the oculomotor system through velocity-pause-saccade neurons, but the activity in these neurons disappears in drowsiness, although the vestibulospinal activity and the rocking persist during drowsiness (36). The velocity storage integrator is under the control of the nodulus in lobule 10 of the vermis of the vestibulocerebellum (40–43). As far as is known, there is no structural or pathologic process that involves the brainstem of those individuals who suffer from the MdDS.

In an accompanying hypothesis (this volume), we postulate that the neurons on both sides of the brainstem oscillate with the activity flowing back and forth, at about 0.2 Hz to activate the body and legs into rocking and swaying at 0.2 Hz. The source of the 0.2 Hz drive is unknown, but it is likely to originate in the nodulus of the vestibulocerebellum, which exerts control over the velocity storage system (42–47). Such activity, which has been found in the cerebellar cortex of the nodulus in the rabbit (48), can be brief or can last for years. Why and how the activity is generated continuously is not understood, but it is clear that the MdDS is an illness without significant pathology that has been determined, at least at this time. Therefore, if appropriate maneuvers or drugs are found to stop the oscillations, subjects can be restored to normality, even after having suffered with the incessant oscillations for many years.

For these reasons, we believe that the other symptoms that accompany the MdDS, i.e., anxiety, brain fog, depression, sensitivity to sound and light, and headaches are likely to be due to the inability to turn off the incessant oscillations. Such symptoms have been widely noted and are exhaustively detailed in a recent article by Van Ombergen et al. (7). Since the process producing the MdDS most likely originates in the velocity storage mechanism in the brainstem, magnetic cortical stimulation is likely to be ineffective in producing long-term relief.

### Limits of the Study

1. We investigated the relationship between self-scored severity and postural instability in patients we have treated successfully (Figure 1). The successful patients had improvement in sway, rocking, and the maximum excursion of the COP of the body. Moreover, the oscillations related to rocking and swaying disappeared in the successful patients. However, the degrees of the improvement in postural stability were lower than the median value of the self-scored improvement that was 78%. Thus, while the postural data are roughly correlated with the tendency of subjective improvement, the quantitative relation is not fully established at present.
2. Because of the widespread origin of the patients, it was impossible to do posturography after the patients left our treatment facility. While the posturography provided an objective measure, there was a portion of patients who did not have a physical sign of instability rather than a sensation of internal motion. Therefore, the measure of the success of treatment at present mainly depends on the patient’s subjective assessment of their status.
3. We used a 10-point scale to assess the overall severity of the MdDS. Although the scale has been commonly used clinically, it has not been verified, and it is not certain how it may have varied among the many patients of different ages, genders, and experiences. At present, there is no standard indexing system to estimate the severity of the MdDS that encompasses all physiological and emotional aspects of the MdDS symptoms. It is certain that a major component should be related to the oscillating sensations, since those are the major features of the MdDS. With a better scoring system, the interindividual difference in reporting the severity may be reduced to allow a better estimate.
4. Although we had a large sampling size, one inadequacy was our failure to have a placebo-controlled crossover design of treatment. Unfortunately, the resources were not available, and it was not possible, particularly in patients who flew to New York from the Midwest, Florida, the West Coast, Canada, Europe, or Australia and spent a week in New York to accomplish the extra study that is likely to be unsuccessful in relieving the patients’ symptoms and signs.
5. Sea legs are common for short periods after many experiences on the sea, but it is not clear why the oscillation can persist for months or years in some people, and particularly in women, centered around menopausal age. There is a similar preponderance of women experiencing motion sickness, with activation of the autonomic system, but why there is this gender predominance is not clear.

## Conclusion

Despite the setback from the initial effectiveness of the treatment, we are pleased that we still achieved a significant improvement in about 50% of classic and spontaneous MdDS patients after 1 year of observation. Thus, the treatment we developed is reproducible (1). If the reversion problem can be resolved, more than 75% of MdDS patients will benefit from this treatment.

## Ethics Statement

This retrospective human study was performed in accordance with the Helsinki Declaration of 1975, as revised in 2013 (49) and was approved by the IRB of Mount Sinai School of Medicine with an exemption of informed consent.

## Author Contributions

MD and SY contributed to research design, data collection, data processing, making figures, and writing of this manuscript. BC and CC contributed to research design and writing of this manuscript. SS referred patients for treatment, and contributed to discussion of treatment results and writing of manuscript.

## Conflict of Interest Statement

Patients payed for this treatment to the Department of Neurology Icahn School of Medicine at Mount Sinai.
